# Insecticidal activity of *Vanillosmopsis arborea* essential oil and of its major constituent *α*-bisabolol against *Callosobruchus maculatus* (Coleoptera: Chrysomelidae)

**DOI:** 10.1038/s41598-019-40148-x

**Published:** 2019-03-06

**Authors:** Eridiane da Silva Moura, Lêda Rita D’ Antonino Faroni, José Cola Zanuncio, Fernanda Fernandes Heleno, Lucas Henrique Figueiredo Prates

**Affiliations:** 1 Departamento de Entomologia/BIOAGRO, Universidade Federal de Viçosa, 36570-900 Viçosa, Minas Gerais Brazil; 2 Departamento de Engenharia Agrícola, Universidade Federal de Viçosa, 36570-900 Viçosa, Minas Gerais Brazil

## Abstract

*Vigna unguiculata*, one of the most important legumes, mainly in underdeveloped countries, is susceptible to post-harvest losses in storage by *Callosobruchus maculatus* (Fabricius, 1775) (Coleoptera: Chrysomelidae). The work evaluated the toxicity, inhibition of oviposition, instantaneous rate of population growth (r_i_) and the development of fumigated *C. maculatus* with the essential oil of *Vanillosmopsis arborea* and its major constituent, *α*-bisabolol. The experimental units consisted of 0.8 L flasks treated with concentrations of 1.2–11.2 μL L^−1^_of air_ of the essential oil of *V. arborea* or its major constituent applied to disks of filter paper. *α*-Bisabolol was quantified as 409.33 mL L^−1^ of the essential oil. The development rate of *C. maculatus* was evaluated by daily adult counts. Oviposition was evaluated at lethal concentrations (LC_50_, LC_25_, LC_10_ and LC_1_). The LC_50_ and LC_95_ of the essential oil of *V. arborea* and *α*-bisabolol were 5.23 and 12.97 μL L^−1^ of air and 2.47 and 8.82 μL L^−1^ of air, respectively. At some concentrations, the *α*-bisabolol was more toxic to males than to females of the insect. Increased concentrations of the essential oil reduced the r_i_, rate of development, oviposition, and number of eggs of *C. maculatus* and therefore have potential for pest control.

## Introduction

*Callosobruchus maculatus* (Fabricius, 1775) (Coleoptera: Chrysomelidae), the main cowpea insect pest^1^, shows cosmopolitan habit, is found on stored legumes and its biology and ecology have been studied^2^. Seed destruction by this insect is often so great that the grains become unfit for human consumption and nonviable for replanting or commercialization^2^ after a few months of storage^3^. Adequate storage of agricultural products aims to reduce losses due to insect damage using mainly chemical control with pyrethroids, organophosphates or fumigants such as phosphine (with aluminum phosphide being its precursor)^4^. Overuse of synthetic insecticides causes risks, toxicity to human health and environmental contamination^5^. Insecticides of plant origin have been investigated to manage insects in stored grains^6–8^. Natural products derived from plants with biologically active compounds^9^ were the first preservatives used by man, originally in their natural state and later on, as oils obtained through distillation in water^10^.

Essential oil of *Vanillosmopsis arborea*, a medicinal plant native to the Araripe National Forest, Ceará, Brazil, is rich in *α*-bisabolol with antibacterial, antifungal and anti-inflammatory activity^11^. *α*-Bisabolol, used in dermatological products^12^, is a sesquiterpene with antiseptic and mutagenic/antimutagenic properties^13^, and antimicrobial activity against *Escherichia coli* and *Staphylococcus aureus*^14^.

The objective was to evaluate the toxicity, oviposition inhibition, instantaneous population growth rate (r_i_) and the development of *C. maculatus* treated with fumigation using *Vanillosmopsis arborea* essential oil and its major component *α*-bisabolol.

## Results

### Essential oil constitution

**Figure 1:**
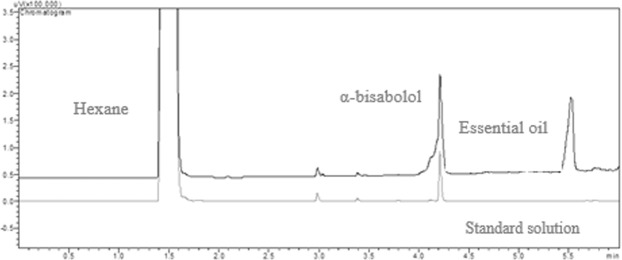
Chromatograms of *Vanillosmopsis arborea* essential oil (200 μL L^−1^ in hexane) and the standard solution of *α*-bisabolol (100 μL L^−1^ in hexane).

The *α*-bisabolol (C_15_H_26_O) was the major component of the essential oil (Fig. 1) and it was quantified by the retention time of 4.2 min at a concentration of 409.33 mL L^−1^.

### Lethal concentrations

The lethal concentrations (LC) required to kill *C. maculatus* adults differed between the essential oil and *α*-bisabolol. The LC_50_ of 2.47 μL L^−1^ and the LC_95_ of 8.82 μL L^−1^ of air of *α*-bisabolol were more lethal than those of the *V. arborea* essential oil with LC_50_ of 5.23 μL L^−1^ of air and LC_95_ of 12.97 μL L^−1^ of air. The concentration-mortality curve slope for the *α*-bisabolol was lower (2.97 ± 0.71) than for the *V. arborea* essential oil (4.17 ± 0.39). The high chi-squared (χ2) and low P (<0.05) values indicate adequacy of the data to the PROBIT model to estimate time-mortality curves (Table 1).

**Table 1:** Relative toxicity of *Vanillosmopsis arborea* essential oil and its *α*-bisabolol component to *Callosobruchus maculatus* (Coleoptera: Chrysomelidae).

Components	LC_50_	LC_95_	Inclination (±MSE^1^)	*X*^2^ (d*f*)	*P*
Essential oil	5.23	12.97	4.17 ± 0.39	10.16 (6)	<0.0001
*α*-Bisabolol	2.47	8.82	2.97 ± 0.71	49.14 (6)	<0.0001

LC = lethal concentration (μL L^−1^ of air); ^1^MSE = mean squared error; *Χ*^2^ = Qui-square; P = Probability; df = degrees of freedom.

### Mortality

*C. maculatus* mortality from *V. arborea* essential oil was lower than that of its major component *α*-bisabolol in almost all concentrations except for 1.2, 1.6, and 11.2 μL L^−1^ air, which were similar to each other. The *α*-bisabolol was more efficient and caused higher *C. maculatus* mortality in five of the eight concentrations tested (2.0 to 8.4 μL L^−1^ of air) (Table 2). When analyzing the mortality of male and females of *C. maculatus*, it was noticed that the mortality of males at concentrations of 1.2, 1.6, and 8.4 μL L^−1^ air was similar between *V. arborea* essential oil and its major constituent. *α*-Bisabolol was more toxic to male at the other concentrations. On the other hand, the female mortality with *V. arborea* essential oil and *α*-bisabolol was similar in low (1.2 and 1.6 μL L^−1^ of air) and high concentrations (5.6, 8.4, and 11.2 μL L^−1^ air). *α*-Bisabolol were more toxic to female at the other concentrations (Table 3).

**Table 2:** Mortality of *Callosobruchus maculatus* (Coleoptera: Chrysomelidae) by *Vanillosmopsis arborea* essential oil and its *α*-bisabolol component at concentrations of 1.2, 1.6, 2.0, 2.4, 5.6, 8.4, and 11.2 μL L^−1^ of air corrected by Abbott’s formula^41^.

Concentration (µL L^−1^)	Essential oil		*α*-Bisabolol	
	Number of dead insects	E (%)	Number of dead insects	E (%)
1.2	5.50 a	22.7	5.00 a	15.0
1.6	6.00 a	29.1	6.25 a	32.0
2.0	6.50 b	34.6	10.75 a	60.4
2.4	7.75 b	45.1	15.50 a	72.5
2.8	8.50 b	50.00	16.50 a	74.2
5.6	14.25 b	70.1	17.75 a	76.0
8.4	17.00 b	75.0	18.25 a	76.7
11.2	18.00 a	76.3	19.00 a	77.6

Means followed by the same letter per line do not differ to a 5% probability by the Tukey test; E (%) = Efficiency of mortality corrected by Abbott’s formula^41^.

**Table 3:** Mortality of females and males of *Callosobruchus maculatus* (Coleoptera: Chrysomelidae) with essential oils of *Vanillosmopsis arborea* and its *α*-bisabolol component at concentrations of 1.2, 1.6, 2.0, 2.4, 2.8, 5.6, 8.4, and 11.2 μL L^−1^ of air corrected by Abbott’s formula^41^.

Concentration (µL L^−1^)	Female				Male			
	Essential oil		*α*-Bisabolol		Essential oil		*α*-Bisabolol	
	Number of dead insects	E (%)	Number of dead insects	E (%)	Number of dead insects	E (%)	Number of dead insects	E (%)
1.2	2.00 a	12.5	2.00 a	12.5	3.25 a	15.3	3.25 a	15.3
1.6	2.25 a	22.2	2.25 a	22.2	3.50 a	21.4	4.50 a	38.8
2.0	2.50 b	30.00	3.25 a	46.1	3.25 b	15.3	7.50 a	63.3
2.4	2.75 b	36.3	5.25 a	66.6	3.50 b	21.4	10.25 a	73.1
2.8	3.25 b	46.1	6.50 a	73.00	4.75 b	42.1	10.00 a	72.5
5.6	5.75 a	69.5	6.00 a	70.0	7.75 b	64.5	11.50 a	76.00
8.4	7.50 a	76.6	7.75 a	77.4	9.75 a	71.7	10.50 a	73.8
11.2	8.75 a	80.0	7.75 a	77.4	10.00 b	72.5	12.25 a	77.5

Means followed by the same letter per line (for female and male separately) do not differ to a 5% probability by the Tukey test; E (%) = Efficiency of mortality corrected by Abbott’s formula^41^.

### Oviposition

The oviposition of *C. maculatus* exposed to *V. arborea* essential oil and *α*-bisabolol was lower than in the control with hexane (Table 4). The decrease in *C. maculatus* oviposition was proportional to the concentration increase, with *α*-bisabolol at LC_50_ responsible for the highest reduction in egg numbers. This confirms the impact of *V. arborea* essential oil and its *α*-bisabolol component on *C. maculatus* oviposition.

**Table 4:** Concentrations used by treatment to evaluate the effect on oviposition of *Callosobruchus maculatus* (Coleoptera: Chrysomelidae) and number of eggs (average ± SD^1^) of *C. maculatus* in cowpea beans treated with *Vanillosmopsis arb*o*rea* essential oil and its *α*-bisabolol component as a function of LC_50_, LC_25,_ LC_10_, and LC_1_.

Treatment	*Vanillosmopsis arb*o*rea e*ssential oil		*α*-Bisabolol	
	Concentration (μL L^−1^ of air)	Number of eggs ± SD^1^	Concentration (μL L^−1^ of air)	Number of eggs ± SD^1^
LC_50_	5.2318	10.75 ± 1.70 aE	2.4705	5.00 ± 0.81 bE
LC_25_	3.6051	35.75 ± 2.21 aD	1.4659	14.00 ± 1.41 bD
LC_10_	2.5784	48.25 ± 2.21 aC	0.9164	23.75 ± 1.5 bC
LC_1_	1.4481	75.00 ± 1.82 aB	0.4083	63.00 ± 1.82 bB
Hexane	—	79.50 ± 1.29 aA	—	79.25 ± 0.95 aA
Control	—	81.00 ± 1.82 aA	—	80.25 ± 1.25 aA

Means followed by the same lowercase letter per line or upper case per column do not differ by a 5% probability by the Tukey test; LC = Lethal concentration (μL L^−1^ of air); ^1^SD = standard deviation.

### Population growth rate

**Figure 2:**
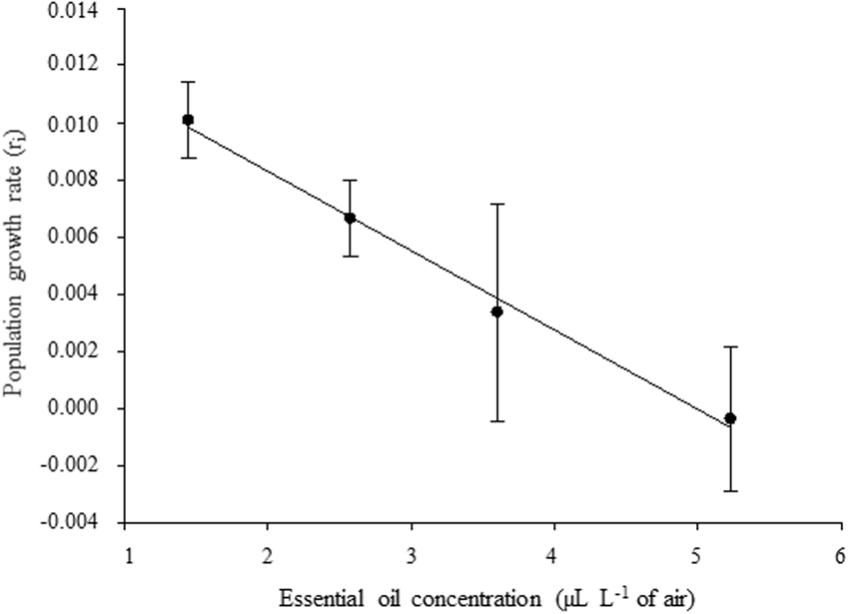
Population growth rate of *Callosobruchus maculatus* (Coleoptera: Chrysomelidae) exposed to the essential oil of Vanillosmopsis arborea as a function of LC_1_, LC_10_, LC_25_ and LC_50_ for 45 days.

**Figure 3:**
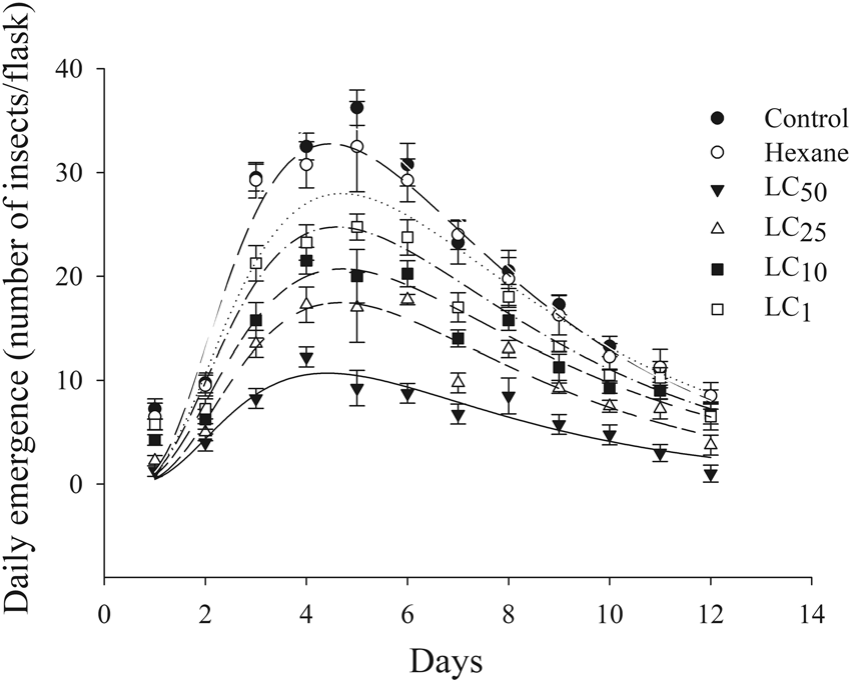
Daily emergence of *Callosobruchus maculatus* (Coleoptera: Chrysomelidae) exposed to the fumigant effect of *Vanillosmopsis arborea* essential oil, to solvent hexane at concentrations LC_1_ (1.4481 μL L^−1^ of air), LC_10_ (2.5784 μL L^−1^ of air), LC_25_ (3.6051 μL L^−1^ of air) and LC_50_ (5.2318 μL L^−1^ of air) and to the oil or solvent (control) for 45 days.

The instantaneous *C. maculatus* population growth rate (r_i_) decreased with increasing *V. arborea* essential oil concentrations (r^2^ = 0.81, F_1.27_ = 113.5, *P* < 0.001) (Fig. 2). The daily emergence of *C. maculatus* adults exposed to *V. arborea* essential oil was maximal between four and six days after emergence onset. The normal Log model was the best fit to describe *C. maculatus* daily emergence exposed to *V. arborea* essential oil. *C. maculatus* daily emergence with *V. arborea* essential oil was higher between the fourth and fifth days after application. The number of adults emerged was higher in the LC_1_ (0.5106 μL L^−1^ of air) and LC_10_ (2.5784 μL L^−1^ of air) of this oil. The minimum *C. maculatus* daily emergence was recorded between 12 and 15 days, with lower values for insects exposed to LC_50_ (5.2318 μL L^−1^ of air). The maximum daily emergence of the insects in the control was between the fourth and sixth days, similar to that of other treatments (Fig. 3).

## Discussion

The chromatographic analysis of *V. arborea* essential oil showed that *α*-bisabolol (C_15_H_26_O) is its main component, as reported for this compound making up to 80 and 80.43% of this essential oil^15,16^. However, factors such as altitude, cultivation, drying conditions, storage, sunshine, temperature, and soil type influence the essential oil composition^17^, explaining the variation in the amount of its compounds.

The LC_50_, 2.47 μL L^−1^ of air to 5.23 μL L^−1^ of air values of *α*-bisabolol and of *V. arborea* essential oil, respectively, are low in relation to those of *Cymbopogon winterianus, Eucalyptus camaldulensis, E. citriodora* and *E. staigeriana*, LC_50_ of 2.58 to 56.7 μL L^−1^ of air^18^ and *E. globulus* essential oil, LC_50_ of 4 μL L^−1^ of air^19^ for *C. maculatus*. The *Heracleum persicum* essential oil presented 219.4 μL L^−1^ of air, for LC_50_ in *C. maculatus* with 12 h exposure^20^ and that of *V. arborea* was lethal for adults of this insect at low concentrations. This oil is a rich source of biodegradable non-toxic bioactive compounds and is potentially suitable against stored grain pests^21^.

The *C. maculatus* adult mortality with these essential oils is due to their volatile compounds such as terpenoids^22^. The effectiveness of these oils depends on factors such as application surface, composition, ecological conditions, dose or concentration, method of application and extraction, penetration pathway, plant parts, season, and insect species^23^. The toxicity of *V. arborea* essential oil for *C. maculatus* adults agrees with its larvicidal effect for *Aedes aegypti* (Diptera: Culicidade)^24^. Gaseous contact of essential oils is neurotoxic, acting on acetylcholine (ACh) inhibition^33^, and it also affects the lipid bilayer cell membrane, the respiratory system, and energy production^16^ increasing its toxicity for insects.

The lethality of *α*-bisabolol at lower concentrations (LC_50_ and LC_95_) for *C. maculatus* compared to that of *V. arborea* essential oil can be explained by its mechanism of action in the activation of K^+^_ATP_ channels^14^. The biological activities of *α*-bisabolol include antibacterial^14^, scarring^25^, mutagenic/antimutagenic activity^13^, inhibition of mast cell granulation^26^, inhibition of the human P450 system^27^, and protection against gastric toxicity induced by acetylsalicylic acid^28^. The *α*-bisabolol at concentrations of 0.5 to 2 mM increased the permeability of the plasma membrane of *Staphylococcus aureus* and *Escherichia coli*^14^. *α*-Bisabolol antimicrobial activity seems to be related to the selective inhibition of ergosterol biosynthesis, antifungal in its pure form or the main component to develop antifungal drugs^29^.

The lower *C. maculatus* female mortality from *V. arborea* essential oil than with its main component agrees with findings for females of this insect exposed to *Ocimum gratissimum* essential oil, being lower than with its main components^30^. This can be explained by sexual dimorphism, with females having a larger abdomen and, therefore, being more resistant to these components^31^. The higher lethality of *α*-bisabolol for *C. maculatus* females may be due to their greater susceptibility in the first days after their emergence, when *C. maculatus* adults begin mating and the female suffers lesions in its reproductive tract, from spikes on the male genitalia^32^. In addition, the period of greatest egg laying occurs one to two days after female emergence, increasing their susceptibility during the first days of adult life to *α*-bisabolol acting on acetylcholine (ACh) inhibition^33^.

The proportional reduction of egg numbers per *C. maculatus* female with increasing concentrations of *V. arborea* essential oil and *α*-bisabolol agrees with findings for *Melia azedarach* L., *Sapindus saponaria* L., *Piper tuberculatum*, and *Sapindus saponaria* L. on *C. maculatus* oviposition^34^, but this activity depends on the plant part from which the extracts were obtained^34^. This behavior may be associated with the secondary substance levels in different plant parts^17^. However, *C. maculatus* survival and oviposition were similar to that with *Amburana cearensis*, *Anadenanthera macrocarpa, Aspidosperma pyrifolium, Cleome spinosa, Croton sonderianus, Hyptis suaveolens, Mimosa tenuiflora, Senna occidentalis*, and *Ziziphus joazeiro* powders^35^. *C. maculatus* oviposition varied with different doses of the *Eucalyptus citriodora* essential oil, from 48.40% to 0.5 mg/100 mg thereof ^36^. Exposure to *Eucalyptus camaldulensis* and *Heracleum persicum* oils reduced *C. maculatus* oviposition^20^. The reduction in the number of eggs per *C. maculatus* female with *V. arborea* essential oil and its component *α*-bisabolol in the LC_50_ can be explained by the higher susceptibility of those mated to monoterpenoids^37^.

Reduction in the instantaneous *C. maculatus* population growth rate (r_i_) with increasing concentrations of the *V. arborea* essential oil agrees with reports for *Zabrotes subfasciatus* (Boh.) (Coleoptera: Chrysomelidae) on bean grains treated with ethanolic extract of *Croton urucurana* leaves^38^. The larvicide effect of *V. arborea* essential oil against *Aedes aegypti* (Diptera: Culicidae) was due to the main constituents of this oil acting individually or synergistically with other constituents^24^ and explains the reduction in the *C. maculatus* population growth rate (r_i_). The barrier effect of the essential oil, together with the lack of respiratory activity and accumulation of toxic metabolites, could explain the death of eggs reducing the population growth rate (r_i_) and development of *C. maculatus* in cowpea beans^39^. In addition, penetration of the oil into the insect egg causes a direct toxic effect, delaying adult emergence and causing adverse effects on the progeny^40^.

*V. arborea* essential oil is rich in *α*-bisabolol and may be an alternative for the the management of *C. maculatus* and other insect-pests in stored products. This oil has a fumigating insecticidal effect on adults reducing female oviposition, population growth rate, and development of this insect. The pure major constituent of *V. arborea* essential oil, the *α*-Bisabolol, caused higher mortality of *C. maculatus* males than *V. arborea* essential oil with a mortality proportional to the concentration increase.

## Methods

### Insects

Adult *C. maculatus* were obtained from the municipality of Crato, Ceará State, Brazil in 2014. These insects were kept in 1.5 L glass flasks with *Vigna unguiculata* cv. always-green grains with moisture content of 10.7% wet basis (w.b.). These flasks were kept in an air-conditioned room at a temperature of 27 ± 2 °C, relative humidity of 75 ± 5% and a 12 h photo period.

### Essential oil

The *Vanillosmopsis arborea* essential oil was obtained by hydrodistillation in a five-liter capacity Clevenger in the Laboratory of Product Technology of the Agricultural Sciences Campus of the Federal University of Cariri. The plant material (leaves, branches and wood) was collected at the Araripe National Forest in Ceará, Brazil (7°19′52.0″S, 39°26′15.9″W). The material was placed in a round bottom flask immersed in 1.5 L distilled water. The extraction time was 2 h, time taken for the oil to accumulate in the water in the condenser, subsequently being separated and stored at 4 °C.

### Chromatographic analysis

The concentration of *α*-bisabolol in the *V. arborea* essential oil was analyzed by Shimadzu GC2010 gas chromatograph (Tokyo, Japan) equipped with flame ionization detector (FID) and DB-5 capillary column (30 m × 0.25 mm × 0.25 μm). The GC configurations were an initial column temperature of 100 °C increasing from 30 °C min^−1^ until 280 °C was reached, the injector temperature was set at 220 °C, and the detector temperature was set at 300 °C. The separation rate of the samples (1.0 μL) injected was 1:5 with nitrogen as the carrier gas and a flow rate of 1.2 mL min^−1^. The concentration of *α*-bisabolol in the essential oil was determined based on the calibration curve constructed from injections of the analytical standard of *α*-bisabolol purchased from Engetec (São Paulo, Brazil) with purity of 99.9%. The total run time was 6 min.

### Fumigation of *V. arborea* essential oil on *C. maculatus* adults

The bioassays were carried out in 0.8 L glass flasks (8 cm diameter × 15 cm height) with 20 one to two day-old *C. maculatus* each in four replications. *V. arborea* essential oil concentrations ranged from 1.2 to 11.2 μL L^−1^ of air. The working solutions for the essential oils were prepared with the solvent hexane (Quimex, F. MAIA Ltda., Brazil) and applied with a microsyringe (Hamilton, Reno, NV, USA) on filter paper disks with a 4.4 cm diameter placed in Petri dishes (6.5 cm in diameter). These plates were covered with organza-type fabric and placed in the base of the flasks. The pure solvent (hexane) was used as the control. The flasks were closed with a screw cap and sealed with parafilm (PM996, American, NV, USA), after the insects were distributed, to prevent oil vapor from leaking during the exposure period. The flasks were kept in a climatic chamber (Model 347 CD, Fanem, São Paulo, Brazil) at 27 ± 2 °C for 48 h. After this period, dead and living insects were counted. This procedure was also performed to evaluate the toxicity for *C. maculatus* males and females with 10 adult insect couples per sample. The average number of dead insects was corrected to adjust their natural mortality and to calculate the efficacy of the essential oil and their respective components by Abbott’s formula^41^.

### Fumigation of *α*-bisabolol on *C. maculatus* adults

Pure *α*-bisabolol was purchased from Engetec (Engenharia das Essências, Brazil). Toxicity assays for LC_50_ and LC_95_ were performed at the 1.2 to 11.2 μL L^−1^ concentrations. Each filter paper disc (4.4 cm) was treated with 25 μL of *α*-bisabolol solution diluted in hexane in a Petri dish (6.5 cm in diameter) covered with organza and inserted into the base of glass flasks with 0.8 L capacity. Twenty unsexed and 20 sexed *C. maculatus* adults (toxicity for males and females) were placed per flask to expose the insects to the fumigant activity of the compounds for 48 h. Each treatment had four replications. The control had 25 μL of pure hexane.

### Effect of *V. arborea*/*α*-bisabolol essential oil on *C. maculatus* oviposition

The fumigant effect of *V. arborea* essential oil and its isolated component on *C. maculatus* oviposition was studied with a lethal concentration and three sub-lethal ones per treatment (LC_50_, LC_25_, LC_10_ and LC_1_) (Table 4). The control had untreated grains. A total of 25 μL of each oil solution, component and solvent (hexane) was applied on filter paper discs (diameter of 4.4 cm). These discs were placed in Petri dishes (6.5 cm diameter) covered with organza and placed on 100 g of cowpea in glass vials (0.8 L capacity). Ten pairs of *C. maculatus*, with one-day emergence, were added to each flask. The insects were kept in a climatic chamber at 27 ± 2 °C. After 48 h, the insects were removed and the number of eggs on the beans counted.

### Effect of *V. arborea* essential oil on the instantaneous growth rate (r_i_) of *C. maculatus* on cowpea

The assay was organized in a completely randomized design with four replications. Each plot was made up of a glass flask (0.8 L capacity) with 100 g of cowpea grains with a moisture content of 10.7% wet basis (w.b.), free of pests and insecticides. The working solutions of the essential oil were prepared with hexane as solvent and applied with a microsyringe (Hamilton, Reno, NV, USA) onto filter paper discs in Petri dishes (diameter 6.5 cm). The Petri dishes were covered with organza-type fabric to prevent direct contact of the insects with the oil and placed on the beans per flask. In the present study, four lethal and sublethal concentrations of the oil, LC_50_ (5.231 μL L ^−1^ of air), LC_25_ (3.605 μL L ^−1^ of air), LC_10_ (2.578 μL L ^−1^ of air) and LC_1_ (1.448 μL L^−1^ of air) were used. The control had untreated grains. The insects were placed in the flasks, which were immediately closed with a screwable metal cap and sealed with parafilm and kept in climatic chambers with a temperature of 30 ± 2 °C and a relative humidity of 70%. After 48 h, the Petri dishes with the essential oil were removed, ending the exposure period for the insects. The metal caps were then removed and the organza flasks returned to the climatic chamber at a temperature of 30 ± 2 °C and relative humidity of 70% for 45 days. The number of live insects and the final weight of the grain mass were evaluated after this period. The instantaneous growth rate of the insects was calculated with the Walthall and Stark equation^42^ (Eq. 1).

1 $${{\rm{r}}}_{{\rm{i}}}=[\mathrm{ln}({{\rm{N}}}_{{\rm{f}}}/{{\rm{N}}}_{0})]/\Delta {\rm{t}}$$

where: N_f_ = final number of insects; N_0_ = initial number of insects and Δt = duration (days) of the test.

### Effect of *V. arborea* essential oil on *C. maculatus* population growth rate on cowpea

Each experimental unit consisted of a glass flask with 0.8 L capacity, containing 100 g of cowpea grains, free of pests and insecticides. The cowpea grains were submitted to treatments in four replications with LC_50_ (5.231 μL L^−1^ air), LC_25_ (3.605 μL L^−1^ air), LC_10_ (2.578 μL L^−1^ air) and LC_1_ (1.448 μL L^−1^ of air) of *V. arborea* essential oil. The control had 25 μL of pure solvent (hexane) and grains without treatment. The solutions of each concentration were applied to the filter paper disks, which were placed in Petri dishes covered with organza, and placed on the grains at the base of the flask. Then, 20 unsexed insects were added to each flask.

The insects were placed in the flasks, which were closed and sealed with parafilm. After 48 h of exposure, the Petri dishes were removed from the flasks, which were covered with organza and packed in a B.O.D. type climatic chamber at 30 ± 2 °C and 70% relative humidity for 15 days. Adult F1 progeny, obtained in the feeding substrate, were counted and removed daily over 45 days from the emergence of the first insect.

### Statistical analyses

The toxicity data was submitted to PROBIT analysis with SAS software (SAS Institute, Cary, NC, USA), generating concentration-mortality curves. The instantaneous growth rate (R_i_) and population development data were subjected to regression analysis using Sigma Plot 12.0 (Systat Software, San Jose, CA, USA). The effect of essential oil or α-bisabolol on oviposition and mortality efficiency were subjected to ANOVA and Tukey’s test with Statistica 8 software (StatSoft Inc, Tulsa, OK, USA).
